# Artificial intelligence in cardiovascular medicine: a practical guide for clinicians and researchers

**DOI:** 10.1093/ehjdh/ztag153

**Published:** 2026-09-24

**Authors:** Christian Basile, Alessandro Villaschi, Pasquale Ambrosino, Mario Enrico Canonico, Araz Rawshani, Emanuele Bobbio

**Affiliations:** Department of Clinical Science and Education, Södersjukhuset, Karolinska Institutet, Stockholm, Sweden; ANMCO Research Center, Heart Care Foundation, Florence, Italy; Department of Clinical Science and Education, Södersjukhuset, Karolinska Institutet, Stockholm, Sweden; Department of Biomedical Sciences, Humanitas University, Pieve Emanuele, Via Rita Levi Montalcini 4, Milan 20090, Italy; Istituti Clinici Scientifici Maugeri IRCCS, Scientific Directorate, Telese Terme Institute, Telese Terme, Italy; CPC Clinical Research, Aurora, CO, USA; Department of Medicine, University of Colorado, Aurora, CO, USA; Departments of Cardiology, Sahlgrenska University Hospital, Blå stråket 3, SE-413 46 Gothenburg, Sweden; Department of Molecular and Clinical Medicine, Institute of Medicine at Sahlgrenska Academy, University of Gothenburg, Bruna Stråket 16, SE-413 45 Gothenburg, Sweden; Departments of Cardiology, Sahlgrenska University Hospital, Blå stråket 3, SE-413 46 Gothenburg, Sweden; Department of Molecular and Clinical Medicine, Institute of Medicine at Sahlgrenska Academy, University of Gothenburg, Bruna Stråket 16, SE-413 45 Gothenburg, Sweden; Inherited and Rare Cardiovascular Diseases, Department of Translational Medical Sciences, University of Campania ‘Luigi Vanvitelli’, Monaldi Hospital, Naples, Italy

**Keywords:** Artificial intelligence, Cardiac imaging, Cardiovascular medicine, Outcome, Machine learning, Precision medicine

## Abstract

Artificial intelligence (AI) is transforming cardiovascular medicine by extracting clinically relevant patterns from complex digital health data, extending beyond traditional statistical approaches. However, the translation of AI from proof-of-concept studies to routine cardiovascular care remains constrained by methodological, ethical, and regulatory challenges. Key barriers include data quality and representativeness, model generalizability, calibration and clinical utility, interpretability, and the risk of bias amplification. To address this gap, this review synthesizes current evidence on AI applications in cardiology, demystifies foundational machine learning concepts for a clinical audience, and outlines a practical framework for best practices in model development, evaluation, and responsible clinical deployment.

## Graphical Abstract

**Graphical Abstract:**
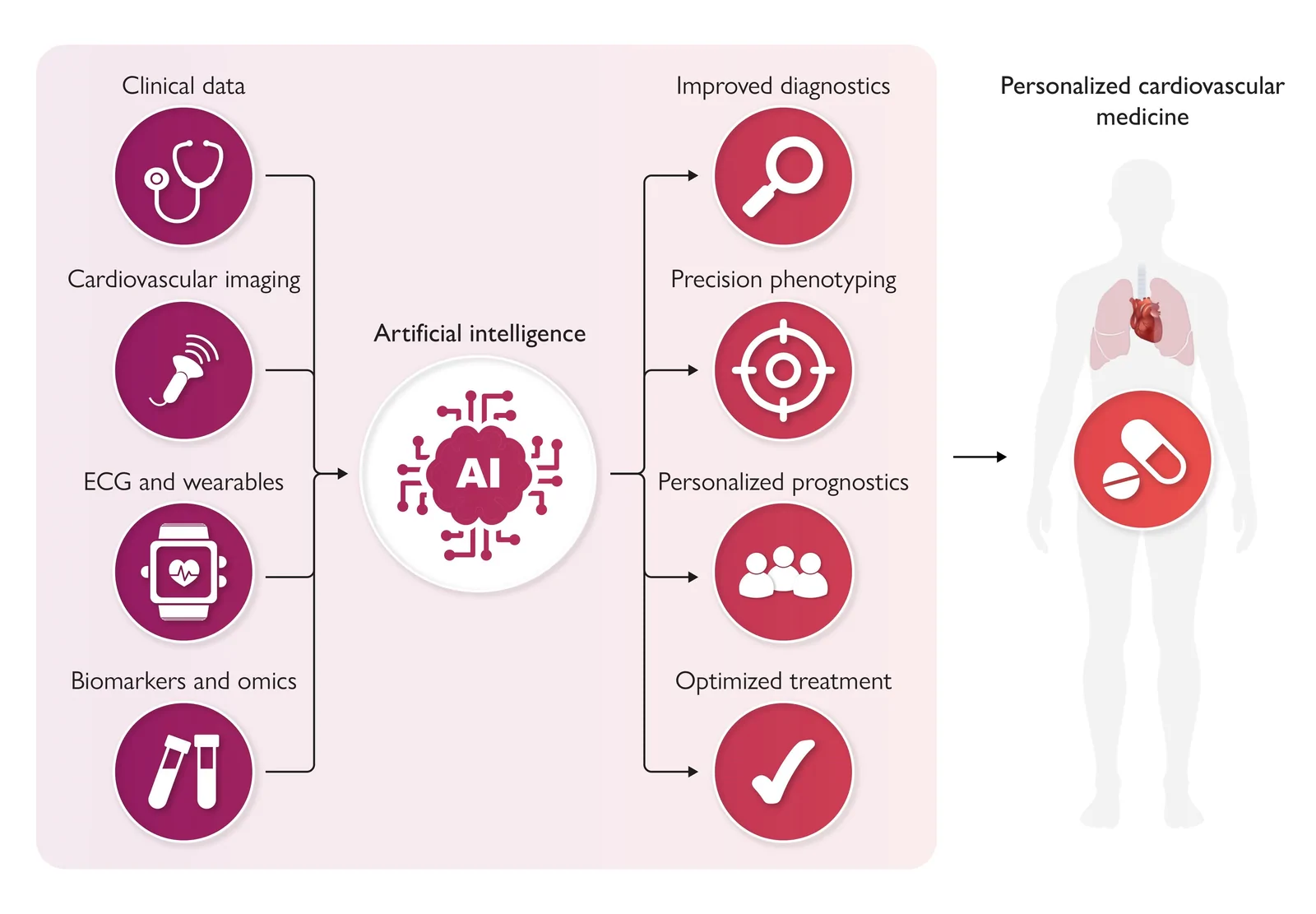
What are we using and where are we heading with artificial intelligence in cardiology?

## Introduction: bridging data science and clinical cardiology

Artificial intelligence (AI) is increasingly recognized as a methodological transformation in cardiovascular medicine rather than a standalone technological innovation.^1^

Cardiology has long been at the forefront of quantitative medicine, with early adoption of electrocardiography, imaging-derived metrics, and multivariable risk scores. However, unlike traditional statistical models, machine learning (ML) architectures learn hierarchical representations directly from raw signals and images, uncovering latent features from complex, high-dimensional datasets.^2,3^

As a result, AI has evolved from experimental applications to tools with growing clinical relevance, demonstrating early success in automating electrocardiogram (ECG) interpretation and reducing inter-observer variability in cardiovascular imaging.^4–7^

Despite these advances, the translation of AI into routine clinical practice remains severely constrained by methodological limitations. Many cardiovascular AI tools are developed using retrospective data from a single institution, leading to significant performance degradation when deployed in diverse, real-world clinical environments due to a lack of external validation.^8^ Furthermore, the opacity of complex algorithms complicates interpretability and clinician trust.^9–11^

To address these challenges, a growing ecosystem of reporting standards and evaluation frameworks has emerged. Updates of established guidelines, including TRIPOD + AI, Consolidated Standards of Reporting Trials extension for AI (CONSORT-AI), and Standard Protocol Items Recommendations for Interventional Trials extension for AI (SPIRIT-AI), provide structured guidance for transparent reporting and risk-of-bias assessment in AI-based clinical studies.^12^ Within this evolving landscape, AI should be understood not as a replacement for clinical expertise but as an assistive tool whose clinical relevance depends on how it is developed, evaluated, and integrated into cardiovascular care (Graphical Abstract). Therefore, the objective of this review is to synthesize current evidence on AI applications in cardiology and demystify foundational ML concepts for a clinical audience.

## Foundations of machine learning: concepts clinicians need to know

### From algorithms to artificial intelligence

The trajectory of AI in cardiology represents the maturation of efforts to quantify cardiac physiology, evolving from early automated rule-based expert systems developed in the 1960s to modern, dynamic approaches. While early heuristic algorithms successfully automated basic ECG interpretation using rigid ‘if-then’ logic, their deterministic structure prevented adaptation to biological variation and limited their robustness to real-world noise.^13,14^

The modern era of AI in cardiology is characterized by a paradigm shift from explicit programming to ML, in which algorithms infer decision rules directly from data. This transition has been driven by the convergence of large-scale digital health data, advances in neural network architectures, and increasing computational power. Today, AI encompasses a broad range of capabilities, from pattern recognition and representation learning to the integration of large multimodal datasets, including demographic variables, biomarkers, and imaging data. Modern AI has transitioned cardiology from instructing computers with predefined rules to empowering algorithms to discover complex, actionable patterns within high-dimensional clinical data, with direct implications for improving diagnosis, risk stratification, and personalized decision-making.^15^

### Common model types and data modalities

The clinical utility of AI in cardiology is fundamentally driven by its ability to integrate diverse data modalities. Cardiovascular datasets are highly heterogeneous and generally fall into four primary categories: structured tabular data (e.g. electronic health records, biomarker panels, and clinical registries), one-dimensional physiological signals (e.g. 12-lead ECGs, Holter monitoring, and wearable telemetry), multidimensional imaging (e.g. echocardiography, cardiac computed tomography, and cardiac magnetic resonance), and unstructured text (e.g. clinical notes and discharge summaries). The choice of ML architecture is intrinsically tied to the specific data modality being analysed.

To understand how these varied data streams are processed, it is essential to define the methodological approaches most commonly used in cardiovascular research. Machine learning methods are broadly categorized into supervised learning, unsupervised learning, and reinforcement learning, each serving distinct roles in clinical investigation and practice (*Figure 1*).

**Figure 1:**
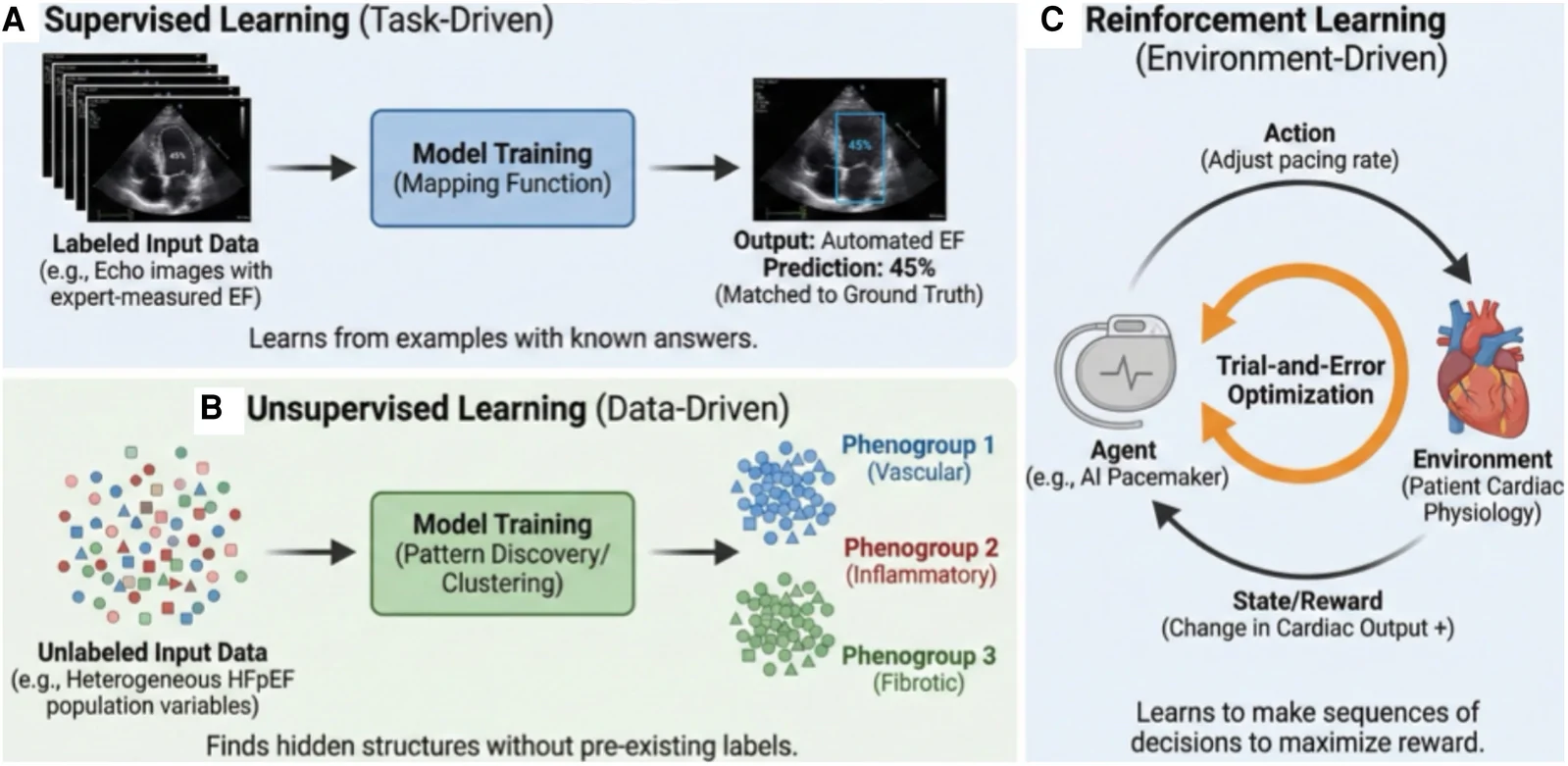
Supervised, unsupervised, and reinforcement learning machine learning models compared. EF, ejection fraction; AI, artificial intelligence; HFpEF, heart failure with preserved ejection fraction.

Supervised learning currently represents the most prevalent approach in cardiology and underpins most AI-based diagnostic tools approved by regulatory authorities. In this paradigm, algorithms are trained using datasets composed of input variables paired with labelled outputs representing the ground truth. The objective is to learn a mapping function capable of predicting outputs for previously unseen data. Supervised learning includes classification tasks, such as identifying heart failure on echocardiography, as well as regression tasks, such as estimating coronary calcium scores from computed tomography images. Deep neural networks, particularly convolutional neural networks (CNNs), have demonstrated strong performance in these applications, especially for imaging-based data.^16^

In contrast, unsupervised learning algorithms operate on unlabelled data and aim to identify latent structures, patterns, or clusters within the dataset. Techniques such as hierarchical clustering and k-means grouping can partition patient populations into phenogroups based on shared characteristics (*Figure 2*). This approach has yielded important insights in heterogeneous clinical syndromes, where traditional classifications may obscure underlying pathophysiological diversity, as exemplified by heart failure with preserved ejection fraction.^9^

**Figure 2:**
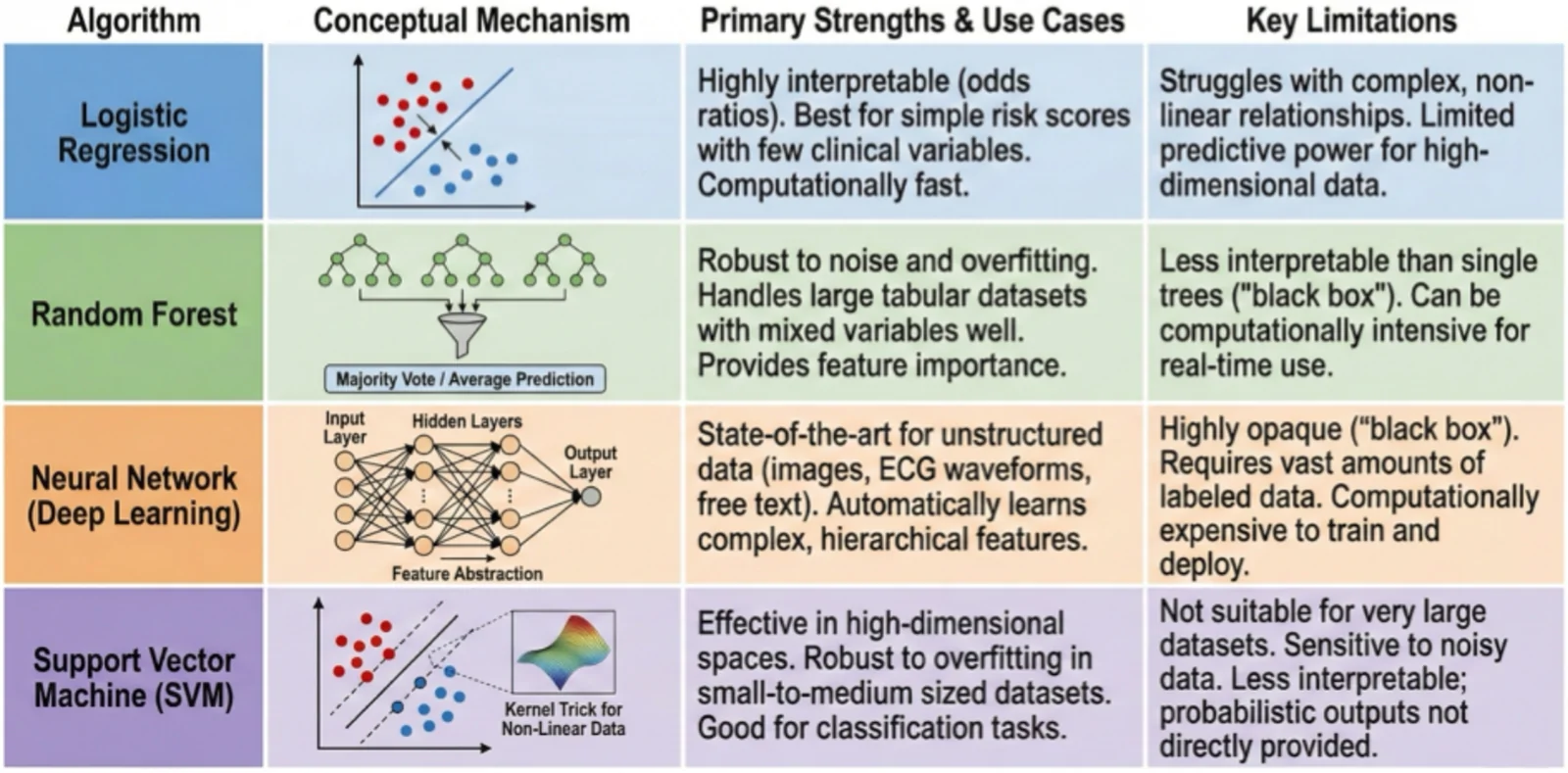
Classic regression algorithms and the most common machine learning algorithms compared. ECG, electrocardiogram.

Reinforcement learning represents a distinct paradigm in which an agent learns to make sequential decisions through interaction with an environment in order to maximize a cumulative reward. Although its application in routine clinical practice remains limited due to the inherent risks of trial-and-error learning in medicine, reinforcement learning has shown early promise in cardiology, particularly for optimizing drug dosing strategies and automating device programming.^17,18^

Within the supervised learning framework, ensemble models constitute a particularly important class of algorithms in cardiovascular medicine. Rather than relying on a single predictive algorithm, ensemble methods combine the outputs of multiple ‘bases’ to improve overall accuracy and robustness. In cardiovascular research, their practical implementation is highly concentrated on analysing structured, tabular clinical data, such as patient registries, biomarker profiles, and multivariable risk predictors, due to their high performance and ability to model non-linear interactions without requiring the massive datasets needed for deep learning (DL) (*Table 1*).^19–21^

**Table 1:** Comparison of machine learning modalities in cardiology

ML approach	Primary clinical use case	Key strengths	Key limitations
Supervised learning	Automated ECG interpretation, imaging segmentation, outcome prediction	High accuracy; straightforward validation; widely accepted by regulatory bodies	Requires massive, manually labelled datasets; susceptible to human annotation bias
Ensemble models	Multivariable risk scores, EHR data analysis	Highly robust to outliers; handles tabular clinical data exceptionally well	Can become a ‘black-box’ compared with single decision tree; requires careful tuning
Unsupervised learning	Phenomapping (e.g. HFpEF subtypes), discovering novel disease clusters	Does not require labelled data; identifies hidden biological heterogeneity	Clinical relevance of clusters is not always clear; highly sensitive to data scaling
Reinforcement learning	Automated device programming, optimizing medication dosing	Can learn optimal, dynamic treatment strategies over time	High safety risk in medicine (trial-and-error,; difficult to validate in traditional RCTs

ECG, electrocardiogram; EHRs, electronic health records; HFpEF, heart failure with preserved ejection fraction; RCT, randomized controlled trials.

To contextualize these algorithms for clinical practice, it is helpful to view them through the lens of technology readiness levels (TRLs).^22^ Supervised and ensemble models frequently operate at relatively high TRLs (e.g. TRL 7–9), with numerous FDA-approved diagnostic algorithms already deployed in routine clinical workflows.^23^ Conversely, unsupervised phenomapping is largely at an intermediate stage (TRL 4–6) and is currently being used to guide clinical trial design and biological discovery. Reinforcement learning remains predominantly at a conceptual or early experimental stage (TRL 2–3) due to the inherent patient safety risks of trial-and-error learning in live clinical environments.^24^

### Limitations of machine learning approaches

One of the primary methodological challenges in developing clinical ML models is the bias-variance trade-off, which directly influences a model’s ability to generalize to new patient populations. Bias reflects errors introduced by overly simplistic assumptions that fail to capture the complexity of the underlying data, leading to underfitting. Conversely, variance reflects excessive sensitivity to noise or idiosyncrasies in the training dataset, resulting in overfitting.^25^

In cardiology, the risk of overfitting is particularly relevant given the relatively limited size of many annotated clinical datasets. For example, a DL model trained to detect hypertrophic cardiomyopathy using data from a single tertiary centre may inadvertently learn scanner-specific artefacts or centre-dependent patterns rather than disease-related features.^25^ To actively mitigate overfitting during model development, researchers must implement methodological safeguards such as cross-validation, data augmentation, and algorithmic constraints during the training phase, rather than relying on *post hoc* validation alone.^26^ External validation then serves as the ultimate independent assessment of whether these safeguards successfully preserved transportability across new populations.^26^ Furthermore, even models that avoid overfitting remain highly susceptible to bias if trained on non-representative populations. For example, a cardiovascular risk prediction algorithm trained primarily on data from a specific, affluent demographic may perform poorly or systematically underestimate risk when applied to diverse ethnic minority populations, thereby exacerbating existing health disparities and potentially leading to inappropriate clinical decision-making among under-represented populations.^26^

Beyond initial deployment, model performance is not static. Algorithms are vulnerable to dataset shift (where the underlying patient population or referral pathways change between the training and deployment environments) and temporal drift (the gradual evolution of disease presentation, diagnostic criteria, or medical treatments over time).^27^ A model that accurately predicts mortality today may become obsolete in 5 years as clinical guidelines evolve, underscoring the necessity of continuous, real-world performance monitoring.

Beyond technical performance, the interaction between clinicians and AI systems introduces significant human–computer interaction challenges and psychological biases. Automation bias may lead clinicians to an over-reliance on AI recommendations, whereas algorithmic aversion may result in the rejection of accurate outputs when model reasoning is not transparent. Addressing these challenges requires explainable AI interfaces that support clinical interpretability and enable clinicians to critically appraise model outputs within the broader clinical context. These limitations motivate the need for rigorous study design, validation strategies, and transparent reporting, which are addressed in the following sections.

## Clinically relevant artificial intelligence models?

As outlined above, AI has the potential to transform multiple domains of cardiology. However, generating clinically meaningful evidence requires AI studies to meet stringent scientific standards related to data quality, model development, validation, and regulatory compliance.

### High-quality datasets

Artificial intelligence research relies on the integration of large volumes of data that, particularly in cardiology, originate from heterogeneous sources such as imaging, electronic health records, free-text clinical notes, and biomarkers.^28^ While data linkage enables the creation of large datasets, it may also consolidate or exacerbate existing biases and limit model performance.^29^ To avoid the ‘garbage-in, garbage-out’ phenomenon, where poor data quality yields unreliable models, a minimum set of quality criteria must be satisfied.

Six key dimensions of data quality have been described: accuracy, completeness, consistency, validity, integrity, and uniqueness.^30^ Accuracy reflects the extent to which data capture the true clinical state and may be compromised by diagnostic coding errors or variable mislabelling. Completeness refers to the proportion of missing data, with high levels of missingness negatively impacting model performance. Consistency, integrity, and uniqueness relate to appropriate linkage across data sources, absence of contradictions, and avoidance of duplicate records. Validity requires that data conform to biologically plausible values and patterns.

Standardized terminologies, careful data preprocessing, and transparent documentation of data handling steps are essential to mitigate these challenges. Data preprocessing, including cleaning, transformation, and integration, facilitates analysis but may itself influence model performance, underscoring the need for transparency. Finally, careful population selection is necessary to avoid exacerbating health disparities and under-representation of minorities.^31,32^

Given the complexity of these processes, interpretability and transparency of both the data pipeline and the resulting models are critical to ensuring trust and accountability.^33^ Different approaches can be used to improve interpretability, including the preferential use of some inherently easier to interpret AI techniques,^34^ such as linear regression or decision trees, and the integration of multiple *post hoc* explanation tools to decode complex ‘black-box’ models.^35^

### Model development and validation strategy

Robust model development requires strict separation of data to ensure generalizability to new patients and to avoid overfitting, a condition in which a model achieves excellent performance on the original dataset but shows poor predictive ability when applied to unseen data. Appropriate data splitting, therefore, represents a foundational step in AI research and typically involves partitioning the dataset into three distinct subsets: a training set, a validation set, and a strictly independent test set reserved for final performance evaluation.^36^

Model training is performed on the training dataset through multiple iterative cycles, during which model parameters are adjusted to reduce prediction error. Fine-tuning then occurs on the validation set, where hyperparameters and configuration choices are optimized to balance predictive accuracy and model complexity. Finally, the test set is used to evaluate model performance on new data, providing an unbiased estimate of generalizability.^37^ In many applications, ∼70% of the data is allocated to training, with the remainder split between validation (20%) and test (10%) sets, although optimal proportions may vary depending on dataset size and study design (*Figure 3*).

**Figure 3:**
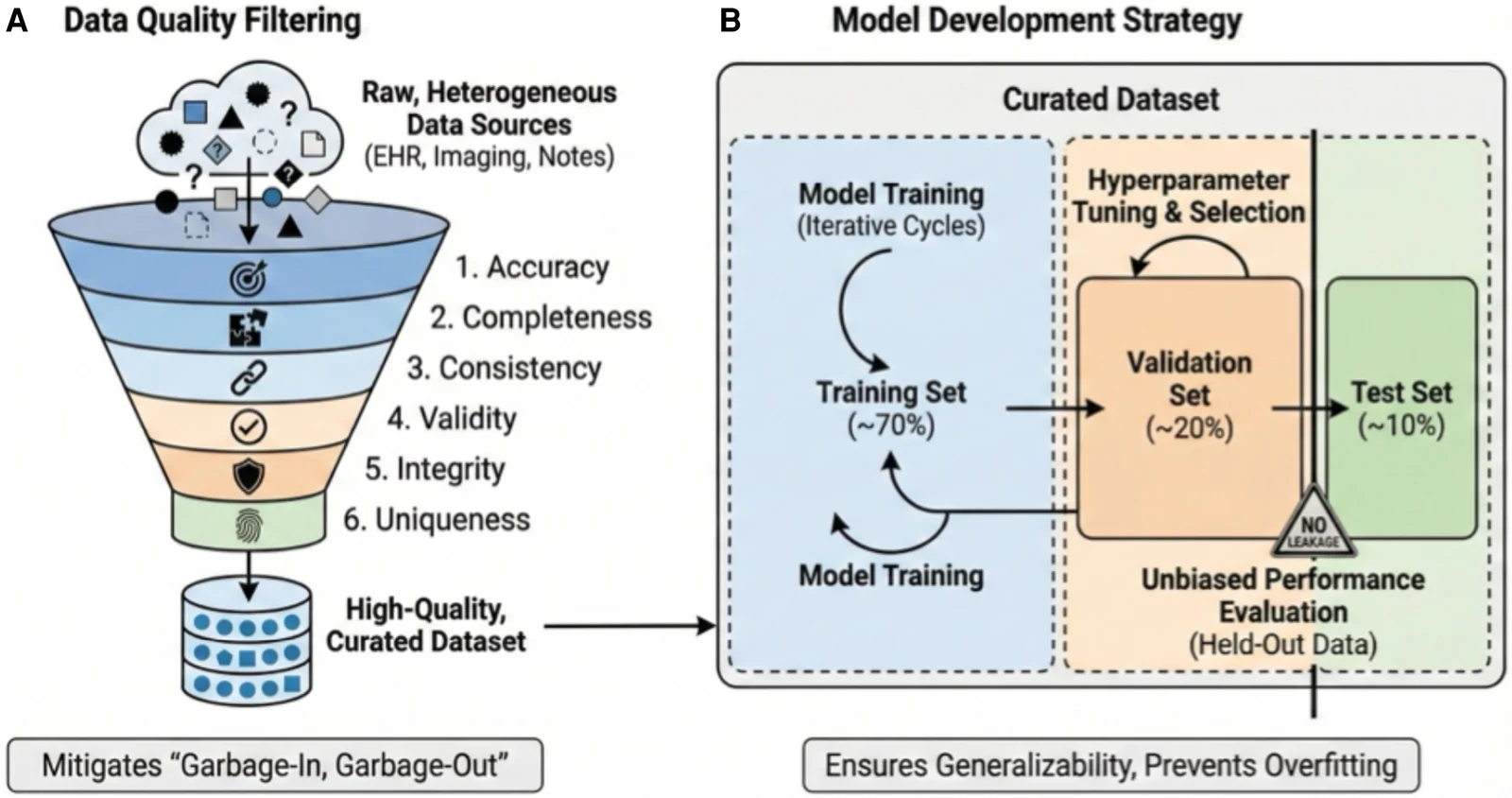
Foundation of artificial intelligence studies: from data quality to model derivation and validation. EHR, electronic health record.

However, while routine data splitting is intended to ensure generalizability, it entails significant methodological trade-offs. The primary limitation of this approach is the inevitable reduction in the effective sample size available for model training. In datasets that are not exceptionally large, withholding 20–30% of the data for validation and testing can severely under-power the learning algorithm and paradoxically increase the risk of overfitting. Therefore, the decision to implement a strict data split must be explicitly justified in relation to the available sample size. Currently, formal sample-size guidance for complex AI architectures in cardiology remains relatively sparse. To address this, cardiovascular researchers should draw on established sample-size frameworks developed for multivariable statistical prediction models. For example, researchers should avoid outdated ‘rules of thumb’, such as requiring a blanket 10 events per predictor variable, which have been shown to be overly simplistic for binary and time-to-event outcomes. Instead, more robust methodologies, such as those proposed by Riley *et al*.,^38^ suggest calculating a minimum sample size designed to meet three specific criteria: (i) ensuring small optimism in predictor effect estimates (targeting a global shrinkage factor ≥0.9), (ii) maintaining a small absolute difference (≤0.05) between apparent and adjusted performance metrics (e.g. Nagelkerke’s *R*^2^), and (iii) ensuring precise estimation of the overall outcome risk in the target population. Applying these rigorous statistical principles is essential to guarantee that an AI model is built on a sufficiently powered dataset before any data partitioning occurs.^38^ Therefore, in specific scenarios where the total available sample size fails to meet these calculated criteria, researchers should avoid rigid data partitioning. Instead, alternative resampling strategies, such as repeated cross-validation or bootstrapping, should be strictly preferred, as they maximize the data available for algorithm training while still providing statistically robust estimates of model generalizability.

Errors may arise at each stage of this process, and ML models remain susceptible to both underfitting and overfitting. In the latter case, cross-validation techniques should be employed to mitigate overly optimistic performance estimates. Although cross-validation includes different methodological variants, its core principle involves rotating training and validation subsets to generate more stable estimates of model performance and robustness, thereby supporting generalizability across different data partitions.^39^

A critical threat to model validity is data leakage, which occurs when information from the test set inadvertently influences the training or validation process. Leakage may arise during feature engineering, normalization, or imputation and leads to artificially inflated performance metrics that fail to replicate in real-world clinical settings. Preventing leakage requires reproducible pipelines that encapsulate all preprocessing and modelling steps, ensuring that data transformations are derived exclusively from the training dataset.^39^

Finally, while internal data splitting and resampling are foundational for model development, they are inherently insufficient to establish true generalizability. A model trained and tested on data from a single institution may still overfit to local idiosyncratic practices, specific patient demographics, or centre-specific imaging equipment. Therefore, validation on a strictly external cohort, comprising data collected from different geographic regions, distinct healthcare systems, or separate time periods (temporal validation), remains the methodological gold standard. External validation is the only robust mechanism to assess an algorithm’s resilience to dataset shift and to confirm its safe transportability into broad, real-world clinical practice.

### Artificial intelligence beyond observational studies and towards randomized clinical trials

To fully answer the question of what makes an AI model clinically relevant, the validation paradigm must eventually extend beyond observational data. Building on the validation strategies discussed in the previous section, it is important to recognize that while retrospective and observational data are sufficient for model development and initial validation, they cannot definitively establish clinical utility. Randomized clinical trials (RCTs) therefore represent the gold standard for evaluating whether AI-driven interventions improve patient outcomes in real-world settings. At the same time, AI methods themselves offer powerful tools to enhance the design, conduct, and efficiency of RCTs, including patient selection, outcome adjudication, data collection, and analysis through techniques, such as ML, DL, and natural language processing (NLP). This dual role highlights the bidirectional relationship between AI and clinical trials in contemporary cardiovascular research.^40^

During the design phase, ML algorithms may be applied to large observational registries to explore how alternative inclusion and exclusion criteria influence patient selection and trial feasibility. These approaches can also support the development of synthetic control arms, reducing the number of patients allocated to placebo, which is particularly relevant for trials involving rare cardiovascular conditions.^41^ Similarly, NLP algorithms enable rapid screening of large datasets to identify eligible patients with higher efficiency than traditional methods, as demonstrated in the COPILOT-HF trial.^42,43^

Beyond recruitment, AI tools may assist in outcome adjudication by extracting relevant clinical information from medical records and standardizing interpretation across sites. Satisfactory performance of these approaches has been demonstrated in heart failure settings, showing high concordance between AI-assisted adjudication and human clinical judgment.^44^ In addition, ML algorithms can be applied during data analysis to identify patient phenotypes or clusters that may respond differently to specific therapies.

Overall, these applications suggest that AI may facilitate RCT design and conduct by improving efficiency, shortening study duration, increasing statistical power, and reducing costs, while remaining complementary to established trial methodologies.^45^

## Evaluating and interpreting artificial intelligence models

### Measuring performance beyond accuracy

In the AI and computer science literature, model performance is frequently reported using threshold-dependent classification measures. These include overall accuracy, sensitivity (recall), specificity, positive predictive value (precision), negative predictive value, and the F1-score (the harmonic mean of precision and recall). While these metrics offer intuitive summaries of a model’s diagnostic ability at a specific decision threshold, their uncritical application in clinical cardiology presents significant limitations.^46^ For instance, overall accuracy can be highly misleading in imbalanced datasets, such as screening for a rare condition, where a model that defaults to predicting the majority class for every patient will mathematically achieve high accuracy while offering no clinical value.^47^ Furthermore, predictive values are inherently dependent on disease prevalence, meaning performance will fluctuate across different cohorts.^48^ Importantly, because these metrics are threshold-dependent, they do not capture the model’s ability to rank patients across the full spectrum of risk and provide no information on calibration, thereby limiting their value for clinical decision-making where absolute risk estimates are required.

Because of these limitations, the evaluation of AI models in cardiovascular medicine requires moving beyond a narrow focus on accuracy or discrimination metrics and adopting a multidimensional assessment framework aligned with clinical decision-making.^49^ Measures such as the area under the receiver operating characteristic curve, together with time-dependent discrimination indices commonly used in survival analysis, remain valuable for describing how effectively a model ranks patients according to risk.^50^ However, discrimination alone does not indicate whether predicted risks are numerically reliable or clinically actionable, particularly in settings where treatment decisions depend on absolute rather than relative risk estimates.^51^

This limitation is highly relevant in cardiovascular care, where thresholds for preventive or therapeutic interventions are often predefined. In such contexts, models may demonstrate acceptable discrimination while still producing systematically biased risk estimates, leading to misclassification of patients around clinically meaningful decision thresholds.^51^ Calibration, therefore, represents a central component of model evaluation. Calibration describes the agreement between predicted and observed risks across the range of clinically relevant probabilities and should be assessed using both numerical summaries, such as calibration-in-the-large and calibration slope, and graphical tools that enable visual inspection of risk agreement.^49^ In cardiovascular applications, calibration assessment is particularly challenging in time-to-event prediction, where differences in baseline hazard, censoring patterns, and competing risks across populations can substantially impair model transportability, even when discrimination appears preserved (*Figure 4*).^52^

**Figure 4:**
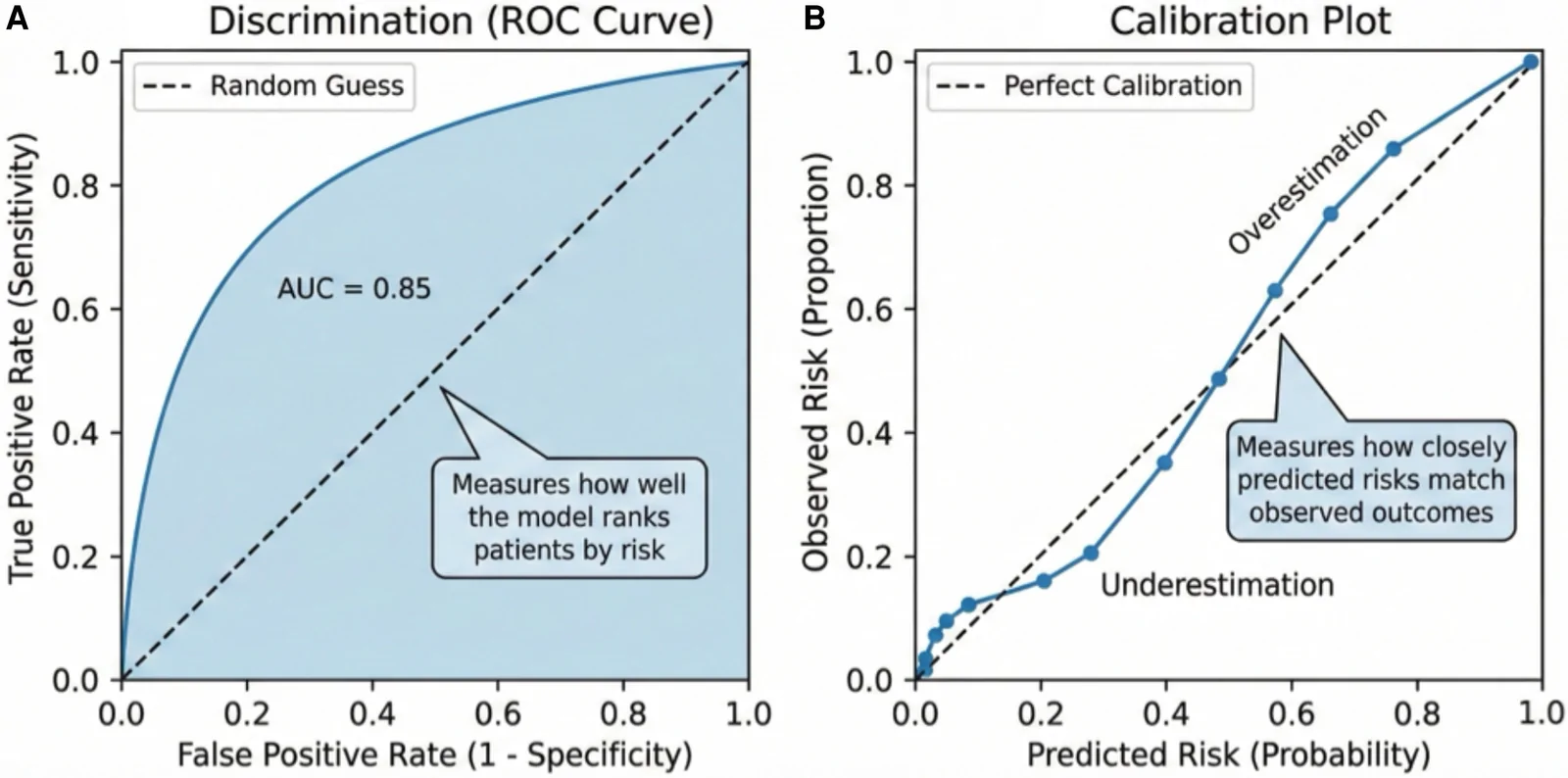
Performance beyond accuracy: discrimination vs. calibration. AUC, area under the curve; ROC, receiver operating characteristic.

As a result, preserved ranking performance may coexist with clinically meaningful miscalibration, with important implications for cardiovascular treatment allocation that are not captured by discrimination metrics alone.^51^ This consideration supports explicit evaluation of calibration drift over time and across clinical settings, rather than reliance on a single static test set as the sole basis for performance assessment.^53^

Beyond statistical performance, evaluation should explicitly address clinical utility.^54^ Decision curve analysis provides a principled framework to quantify net benefit across a range of risk thresholds and to compare AI models with alternative strategies, including guideline-based care or established clinical risk scores.^55^ This approach is particularly relevant in cardiovascular medicine, where intervention thresholds for lipid-lowering therapy, anticoagulation, or device implantation are often specified a priori by contemporary clinical guidelines.^56,57^ By formalizing the trade-off between overtreatment and undertreatment, decision curve analysis links model predictions to clinical consequences and clarifies whether adoption of a model is likely to improve decision-making in practice.^55^

Validation strategies also warrant careful consideration. Internal validation remains essential to quantify optimism and overfitting during model development but is insufficient to support clinical deployment.^58^ External validation, whether temporal, geographic, or across different data acquisition pipelines, probes model robustness under conditions of dataset shift and should be regarded as a prerequisite for credible claims of generalizability.^58^ Performance degradation observed in external cohorts should not be interpreted solely as model failure, but rather as evidence of differences in population characteristics, measurement processes, or cardiovascular care pathways that may limit transportability and may necessitate recalibration, model updating, or restriction of the intended use.^52^

### Interpretability, explainability, and clinician trust

Interpretability and explainability represent distinct concepts in ML. Interpretability refers to the inherent transparency of a model, in which the mathematical mapping from input to output is directly understandable to humans, as in linear regression or simple decision trees. Explainability, conversely, involves applying *post hoc* techniques to decode complex, opaque ‘black-box’ models (such as deep neural networks) into human-comprehensible terms.

In clinical practice, both concepts ultimately serve a unified purpose: the ability to anticipate model behaviour, recognize plausible failure modes, and support accountable decision-making under conditions of uncertainty, rather than as a property of specific analytical techniques.^59^ In cardiology, where AI systems increasingly inform diagnostic interpretation and prognostic stratification, clinicians must be able to judge when model outputs warrant confidence and when caution is appropriate, a judgment grounded in clinical plausibility and contextual understanding at least as much as in numerical performance metrics.^59^ This perspective reframes interpretability from a purely technical requirement into a clinical competency that underpins responsible use of AI at the bedside.

*Post hoc* explanation methods, including Shapley additive explanations and gradient-weighted class activation mapping, can offer insight into which input features or image regions contribute most to individual predictions.^60^ The primary advantages of these methods lie in their utility for model debugging and error analysis. For example, in cardiovascular imaging, visual heatmaps can serve as a crucial ‘sanity check’ to reveal whether a model is improperly relying on spurious correlations, such as identifying a pacemaker or a specific hospital’s text watermark, rather than on actual myocardial pathology. Furthermore, by highlighting non-linear interactions among features, these tools can serve as valuable engines for hypothesis generation in clinical research. However, *post hoc* methods also have significant limitations that clinicians must recognize. First, they often lack strict fidelity to the underlying ‘black-box’ model; the explanation is merely an approximation and may not accurately reflect the model’s true mathematical reasoning.^34^ Second, many of these techniques suffer from instability, where microscopic, clinically irrelevant changes to the input data can drastically alter the resulting explanation. Third, they introduce a high risk of ‘confirmation bias’, wherein a clinician might place unwarranted trust in an incorrect prediction simply because a heatmap highlights a biologically plausible region.^61^

Ultimately, these approaches may enhance transparency and facilitate error analysis, particularly in imaging and electrocardiographic applications, and are valuable tools for model debugging. However, they do not convert complex predictive models into causal or mechanistic explanations.^60^ Consequently, their outputs require cautious interpretation and, where feasible, validation against independent clinical or experimental evidence, to avoid over-interpretation of model-specific artefacts as meaningful pathophysiological insights.^62^

From a practical standpoint, when an AI system’s prediction or its accompanying explainability heatmap directly contradicts a clinician’s well-founded judgment, the clinician should not reflexively defer to the algorithm. The literature consistently demonstrates the risks of ‘automation bias’, wherein physicians may adopt incorrect AI suggestions even when contradictory patient data is available.^63,64^ Therefore, AI discordance should be treated strictly as a trigger for heightened clinical scrutiny, prompting a re-evaluation of the raw diagnostic data, an assessment of patient-specific context or multimorbidity that the model may lack, and a review for potential algorithmic failure modes. Ultimately, clinical sovereignty must prevail; AI remains an assistive adjunct, and the final management decision must always be anchored in a comprehensive, human-led clinical evaluation.

### Reporting standards and frameworks for trustworthy artificial intelligence

Transparent reporting is a prerequisite for meaningful interpretation, comparison, and synthesis of AI studies in cardiovascular medicine. In contrast to methodological rigour, which depends on study design, data quality, and validation, reporting standards define how evidence is communicated, enabling readers to assess what was done, under which assumptions, and within which clinical context.^65^ This distinction is particularly relevant for AI-based studies, where complexity and opacity can obscure critical methodological choices if not explicitly documented. The recently published FUTURE-AI international consensus guideline conceptualizes trustworthy healthcare AI across six distinct principles: Fairness, Universality, Traceability, Usability, Robustness, and Explainability.^66^ Under this framework, concepts that are often improperly conflated with explainability are given distinct operational domains. For example, the transparent reporting of data provenance, the handling of missing data, and continuous algorithmic auditing fall under the principle of Traceability. Traceability ensures accountability by requiring comprehensive technical and clinical documentation throughout the AI lifecycle, enabling clinicians and regulators to monitor the tool’s ongoing safety. Similarly, Fairness and subgroup performance analyses represent a distinct pillar. Differences in error rates or calibration across demographic and clinical subgroups must be actively evaluated and mitigated to fulfil the Fairness principle, ensuring equitable performance across diverse populations. Fairness and subgroup performance analyses also contribute to clinician trust but require careful framing.^67^ Differences in error rates or calibration across demographic or clinical subgroups should be examined when clinically justified and interpreted in relation to downstream clinical consequences, including the potential amplification of existing inequities.^67^ Importantly, Fairness does not imply identical performance across all subgroups. Exploratory subgroup analyses should therefore be explicitly labelled as such to avoid unwarranted claims of equity or false reassurance regarding model performance across populations.^68^

Several reporting and appraisal frameworks have been developed to address these challenges. The Transparent Reporting of a multivariable prediction model for Individual Prognosis Or Diagnosis extension for AI (TRIPOD + AI) provides structured guidance for reporting prediction model development and validation, with explicit attention to data sources, preprocessing decisions, model specification, and performance assessment.^65^ Importantly, TRIPOD + AI should be interpreted strictly as a transparency framework rather than as a surrogate marker of methodological quality, a distinction that remains underappreciated and contributes to misinterpretation of AI evidence in the literature. Complementing this, the Prediction model Risk Of Bias ASsessment Tool extension for AI (PROBAST + AI) offers a structured approach to evaluating risk of bias and concerns regarding applicability, particularly in the context of systematic reviews and evidence appraisal.^69^

When AI systems are evaluated within randomized or quasi-randomized interventional studies, the CONSORT-AI and the SPIRIT-AI define essential elements for transparent reporting, including the role of the system within the clinical pathway and its interaction with human decision makers.^70,71^

For early phase evaluation and real-world implementation, the Developmental and Exploratory Clinical Investigation of Decision support systems driven by AI (DECIDE-AI) framework addresses reporting needs that are not captured by traditional trial or prediction model guidelines.^10^ By focusing on feasibility, safety signals, and human–system interaction in live clinical environments, DECIDE-AI provides a structured approach to documenting transitional phases between retrospective validation and routine clinical use.^10^ This perspective is particularly relevant in cardiology, where workflow integration and clinician behaviour may substantially influence observed outcomes.

Across these frameworks, a unifying principle is explicit delineation of scope and context (*Figure 5*). Clear reporting of intended use, validation setting, and assumptions underlying model deployment allows readers to judge transferability and limits of applicability without conflating transparency with performance claims. From a clinical standpoint, the most defensible contributions are those that clearly situate AI systems within a defined evidentiary context and present deployment as a testable and revisable intervention rather than as a static product.^10,53^

**Figure 5:**
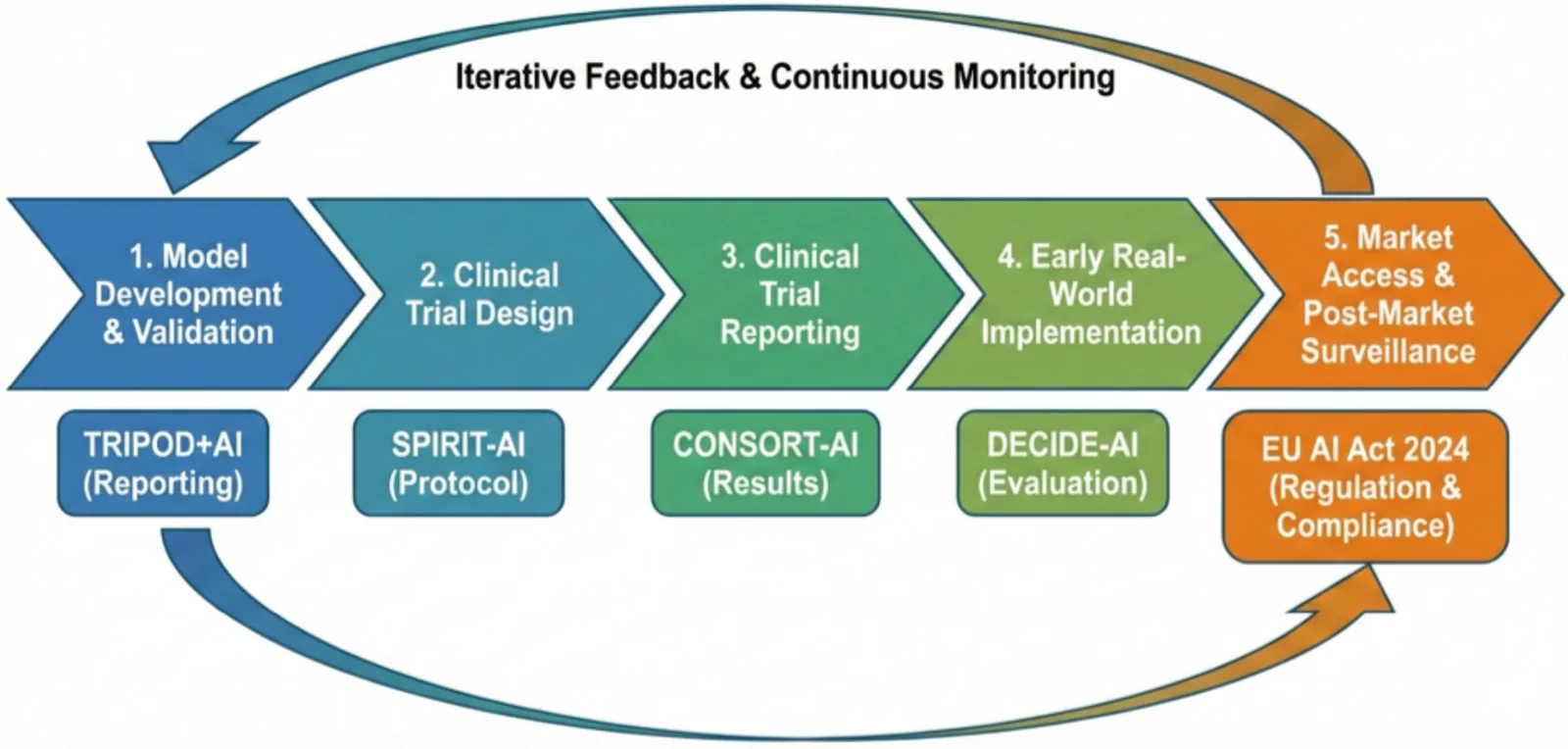
Reporting and regulatory roadmap for the use of artificial intelligence in medicine.

## From model to medicine: clinical applications

### Imaging and diagnostic support

The application of ML in cardiovascular imaging is reshaping the workflow of diagnosis and risk stratification. Echocardiography, the primary cardiac imaging modality, has historically exhibited significant inter-observer variability and dependence on sonographer skill. Deep learning models, such as the U-Net architecture, are now capable of fully automated view classification and segmentation of cardiac chambers. The EchoNet-Dynamic study demonstrated that a video-based DL algorithm could predict ejection fraction with a mean absolute error comparable to the inherent variability between human experts.^6^ These tools can significantly reduce total acquisition and processing time. Beyond simple measurements, AI helps disentangle complex diagnoses where visual assessment is insufficient. For example, in cardiac magnetic resonance, ML has automated quantification of post-infarct scar, distinguishing dense scar from grey-zone fibrosis with high precision.^72^

### Electrocardiogram and arrhythmia prediction

The ECG is the most widely used cardiovascular diagnostic test, and DL has shown that the ECG signal contains a wealth of subvisible physiological information. A landmark application of AI-ECG is the detection of paroxysmal atrial fibrillation in the setting of normal sinus rhythm. When a CNN was trained on nearly 650 000 ECGs to identify a fingerprint of atrial fibrillation that persists even when the patient is in sinus rhythm at the time of recording, it recognized the arrhythmic substrate with high accuracy, directly addressing the clinical challenge of cryptogenic stroke.^4^ Furthermore, the ECG AI-Guided Screening for Low Ejection Fraction (EAGLE) trial demonstrated the efficacy of AI-ECG in screening for asymptomatic left ventricular dysfunction, identifying patients with low ejection fraction with high sensitivity and specificity. Patients with a positive AI screen were found to have a four-fold increased risk of developing future ventricular dysfunction, enabling the repurposing of a low-cost test as a population health screening instrument.^73^ The utility of AI-ECG also extends to structural abnormalities, with algorithms detecting moderate-to-severe aortic stenosis and hypertrophic cardiomyopathy from standard 12-lead traces. The democratization of ECG via wearables has further expanded this frontier, with FDA-cleared algorithms now available on consumer devices to detect arrhythmias, placing a triage tool directly in the patient’s pocket.^74^

### Prognostic modelling and precision medicine

AI algorithms are transforming prognostic modelling by moving beyond traditional risk scores to more personalized assessments. In coronary computed tomography angiography, AI enables the detailed characterization of atherosclerosis by quantifying features associated with plaque vulnerability. Machine learning models can segment and quantify low-attenuation plaque, calcification, and positive remodelling with high precision. Studies such as the Exploring the Mechanism of Plaque Rupture in Acute Coronary Syndrome Using Coronary Computed Tomography Angiography and Computational Fluid Dynamics II (EMERALD-II) have shown that AI-quantified plaque burden is a superior predictor of future myocardial infarction than stenosis severity alone, supporting a shift in management towards treating total plaque burden with aggressive lipid-lowering therapies.^75^ Additionally, technologies like fractional flow reserve computed tomography utilize computational fluid dynamics and DL to model blood flow through a patient’s specific coronary anatomy, creating a digital twin that simulates physiological conditions to determine ischaemia non-invasively.^76^

In the rapidly evolving field of structural heart disease, AI is increasingly utilized to inform complex, multimodal procedural decisions. For instance, in the management of severe aortic stenosis, ML algorithms have been developed to model comparative outcomes between transcatheter aortic valve implantation and surgical aortic valve replacement. Traditional surgical risk calculators, such as the STS-PROM or EuroSCORE II, often demonstrate modest discriminative capacity and poor calibration for patients undergoing transcatheter procedures.^77^ In contrast, AI models integrate multidimensional data, including baseline frailty indices, detailed echocardiographic parameters, and preoperative anatomical features from computed tomography, to more accurately predict peri-procedural complications and long-term mortality.^78^ By providing personalized, patient-specific risk stratifications, these models directly support the multidisciplinary Heart Team in selecting the optimal intervention modality for individual patients.

The most compelling application of AI in precision medicine is the phenomapping of heterogeneous syndromes. Heart failure with preserved ejection fraction has historically been a challenging diagnosis with few effective therapies. Researchers have used unsupervised learning to deconstruct this syndrome by applying clustering algorithms to dense phenotypic data. This approach identified distinct phenogroups with divergent pathophysiology and outcomes, providing a basis for biologically informed stratification. This data-driven taxonomy is now guiding the design of stratified clinical trials, moving cardiology closer to a model where treatment is matched to the specific biological phenotype rather than the generic syndrome.^79^ Finally, reinforcement learning is emerging as a tool for automated therapeutic optimization, showing promise in managing complex dosing protocols for anti-arrhythmic drugs and optimizing the programming of cardiac devices based on continuous physiological feedback.^80^

## Ensuring quality, ethics, and compliance

Ensuring quality, ethical integrity, and regulatory compliance is fundamental for the responsible diffusion of AI in cardiology, a field in which rapid technological innovation directly intersects with patient safety and evolving regulatory requirements. High-quality AI development requires diverse and representative data, together with transparent model design, to mitigate disparities in CV research. Bias in training datasets remains one of the most prominent ethical concerns, alongside the risk of uneven model performance across ethnic, socioeconomic, or geographically distinct populations, potentially disadvantaging vulnerable and under-represented groups.^81^ Data privacy and security represent additional critical challenges, particularly as cardiology increasingly relies on longitudinal data streams derived from wearables, implantable devices, and remote monitoring systems.^82^ These concerns are further amplified by uncertainty surrounding secondary data use, data ownership, and patient consent, reinforcing the need for transparent communication regarding data flows, robust cybersecurity infrastructure, and clearly defined governance frameworks.

Another central ethical challenge relates to the ‘black-box’ nature of many AI systems and its implications for clinician accountability and patient autonomy. Limited interpretability may impair clinicians’ ability to critically assess AI-generated outputs, explain results to patients, and appropriately integrate algorithmic recommendations into shared decision-making processes. In this context, clear, comprehensive, and transparent communication should be regarded not merely as a technical feature, but as an ethical requirement.^83^ This is particularly relevant in cardiovascular (CV) applications such as arrhythmia detection, cardiovascular imaging, and the prediction of peri-procedural outcomes, where misinterpretation of AI-derived results may lead to inappropriate clinical actions with direct consequences for patient safety.

Regulatory compliance frameworks are evolving to address these challenges. Within the European Union, the 2024 Artificial Intelligence Act (AI Act) introduces a cross-sector regulatory system that classifies medical AI applications as ‘high-risk’, thereby subjecting them to stringent requirements for transparency, risk management, and post-market surveillance.^84^ The AI Act identifies safety, transparency, and accountability as core principles governing the use of AI in healthcare. The AI Act also explicitly addresses risks related to algorithmic, data-related, selection, and automation bias, which may contribute to reduced performance in under-represented populations or the overestimation of AI outputs.^84^ Mitigation strategies, including the use of diverse and representative datasets, continuous performance evaluation, and meaningful human oversight, are therefore essential, especially in high-risk clinical settings such as cardiovascular disease. Transparency further requires clear documentation of AI system design, intended use, and limitations, together with appropriate disclosure to clinicians and patients.

While the AI Act provides a critical foundation for the regulation of AI in healthcare, several challenges remain.^85^ These include heterogeneous implementation across European Union member states, uncertainty regarding risk categorization for emerging AI tools, and the need to harmonize the AI Act with existing medical device regulatory pathways.^85^ Addressing these gaps will require coordinated governance, adequate regulatory capacity, and clear accountability, particularly when AI systems contribute to clinical decision-making.

Translating these regulatory requirements and ethical principles into clinical reality requires a robust, phased deployment strategy. Implementing AI is not merely the adoption of a new software layer, but a sociotechnical reconfiguration of clinical workflows. To ensure safety and operational feasibility, algorithms should initially undergo a ‘shadow mode’ or silent deployment, running prospectively on live data to evaluate local calibration without influencing clinical management.^86^ Furthermore, because AI tools that require clinicians to leave their primary workspace routinely suffer from poor adoption, successful deployment necessitates embedding AI insights seamlessly into existing electronic health records and imaging archives.^87^ Finally, because models trained on historical data are vulnerable to performance drift as patient demographics, disease prevalence, or imaging hardware evolve, clinical deployment must be treated as a continuous process.^88^ It mandates rigorous post-market surveillance to detect calibration decay and trigger essential model updates.

The patient perspective represents another key dimension of AI adoption in cardiovascular care. Patients tend to accept AI-supported clinical decision-making only when clinician involvement, transparency, and meaningful interpretation of AI outputs are ensured.^82^ Both patients and clinicians have expressed concerns that commercial incentives, limited algorithmic transparency, and inadequate user support may negatively affect patient-centred outcomes.^89^ Beyond robust regulation and technical validation, clear communication strategies, clinician education, and shared decision-making models are therefore required to ensure that AI functions as an assistive tool rather than a replacement for clinical judgment.^83^

Taken together, ensuring quality, ethics, and compliance in cardiovascular AI requires a coherent ecosystem that integrates rigorous data governance, interpretable algorithms, clinical trial evidence, harmonized regulatory pathways, and ongoing real-world monitoring (*Figure 6*). Patient safety depends on robust anonymization and encryption measures, as well as compliance with the General Data Protection Regulation, which establishes high standards for data security and protection of health information. Meeting these requirements is essential for the ethical development and responsible diffusion of AI, and for maintaining trust among patients, clinicians, and society.^90^ A multidisciplinary approach to AI governance has the potential to improve cardiovascular outcomes while preserving equity, accountability, and patients’ trust.

**Figure 6:**
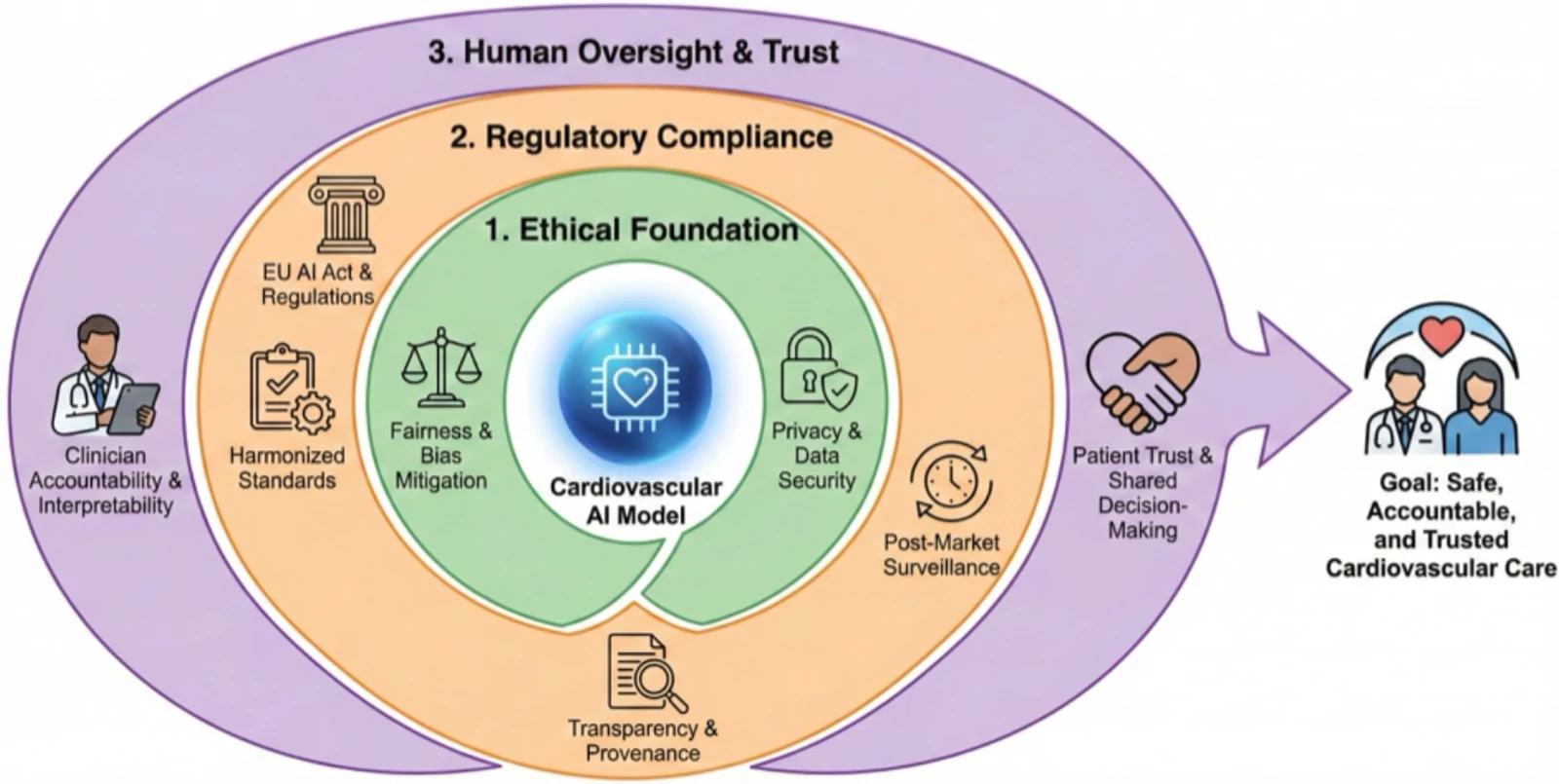
Ethical steps for a responsible use of artificial intelligence in cardiology. AI, artificial intelligence; EU, European Union.

## Conclusions

The central challenge for contemporary cardiology is no longer whether AI can achieve high predictive performance, but how it can be translated into clinically reliable, equitable, and actionable decision support (*Table 2*).

**Table 2:** Key recommendations for the development, evaluation, and integration of AI in cardiology

Area of focus	Key recommendation
Data quality	Ensure training datasets are sufficiently large, diverse, accurate, and properly preprocessed. Careful population selection is necessary to prevent bias amplification and ensure equitable model performance across demographic groups.
Model validation	Internal data splitting is not sufficient for clinical deployment. Independent, external validation across different geographic locations and healthcare systems is a strict prerequisite to confirm generalizability and robustness against overfitting.
Performance evaluation	Move beyond simple discrimination metrics (e.g. AUC). Explicitly assess model calibration (agreement between predicted and observed risks) and clinical utility (via decision curve analysis) to ensure predictions are actionable and reliable.
Reporting and transparency	Utilize established, AI-specific reporting and appraisal frameworks (such as TRIPOD + AI, PROBAST + AI, and DECIDE-AI) to transparently document data provenance, methodological choices, and the intended clinical context.
Clinical interpretability	Recognize that *post hoc* explainability tools do not equate to causal or mechanistic explanations. Clinicians should be trained to critically appraise AI outputs within the broader clinical context to avoid automation bias.
Implementation and monitoring	Treat AI as an assistive tool rather than a replacement for clinical judgment. Implement continuous, real-world post-market surveillance and periodic model recalibration to detect dataset shift and performance degradation over time.

High-quality and representative datasets, model development, and validation across populations and healthcare settings are essential to ensure generalizability and to avoid misleading performance claims.

From a practical perspective, AI is likely to be implemented more widely over time. A clear definition of the intended use, adherence to reporting standards, alignment with regulatory requirements, and post-deployment surveillance are necessary to ensure accountability and patient safety.

## Data Availability

No new data were generated or analysed in support of this research.
